# A NEW ERA IN ASSESSING THE BURDEN OF HEARING LOSS IN LATER LIFE USING PATIENT RECORDS AND IMPLICATIONS FOR HEALTHY AGING

**DOI:** 10.1093/geroni/igae098.0166

**Published:** 2024-12-31

**Authors:** Dialechti Tsimpida

**Affiliations:** University of Southampton, Southampton, England, United Kingdom

## Abstract

Hearing loss is a significant global health concern and a leading cause of disability in later life. While conventionally considered an inevitable consequence of aging, recent evidence highlights modifiable risk factors challenging this notion. Nevertheless, epidemiological estimates, including Global Burden of Disease studies, overlook the actual hearing health needs of populations, relying instead on projected age demographics. This study aimed to provide a fresh perspective, quantifying hearing loss burden and distribution in older adults by analysing anonymised longitudinal medical records of 2.7 million registered patients in the Cheshire and Merseyside Integrated Care System (ICS) in England from 2013 to 2022. Using advanced spatial analysis techniques, including Cluster and Outlier Analysis and Geographically Weighted Regression, socio-spatial inequalities in hearing loss prevalence and incidence were investigated. Surprisingly, findings revealed hearing health disparities unrelated to population age, with residents of similar age experiencing worse hearing outcomes in different areas of the ICS. Deprivation emerged as a significant determinant, explaining up to 35% of the variance in hearing loss. These findings underscore the need for a paradigm shift in health policy-making, moving beyond age-based projections to more accurately estimate and address hearing health needs. Hearing health indicators are already available in existing patient records and should be systematically reported, analysed, and interpreted to create equitable long-term care models according to populations’ actual needs. This novel data-driven approach is particularly important in the context of healthy aging initiatives, given that hearing capacity lies at the core of the Integrated Care for Older People (ICOPE) approach.

